# Graphical reduction and analysis small-angle neutron scattering program: *GRASP*

**DOI:** 10.1107/S1600576723007379

**Published:** 2023-09-20

**Authors:** C. D. Dewhurst

**Affiliations:** a Institut Laue–Langevin, 71 avenue des Martyrs, 38042 Grenoble, France; Argonne National Laboratory, USA

**Keywords:** small-angle neutron scattering, software, data treatment, *GRASP*, MATLAB, multidetector data

## Abstract

*GRASP* is a scientific software application designed for the graphical inspection, reduction and analysis of multidetector data produced by the small-angle neutron scattering (SANS) instruments at the Institut Laue–Langevin and other neutron sources around the world. The first developments of *GRASP* began more than 20 years ago and were written in MATLAB, allowing rapid development of scientific code, with much of the data handling, matrix manipulation, mathematical tools, user interface and graphical tools integrated at a high level in the underlying MATLAB platform. By their very nature, multidimensional data are often best appreciated in graphical form. *GRASP* deals with many of the diverse requirements for data reduction and analysis of SANS data using a general set of tools and reduction algorithms suited to 2D multidetector data. A further fundamental architectural inclusion is a third dimension of data manipulation, thereby easily allowing parametric analysis and cross referencing of series data such as composition, kinetic measurements, temperature, magnetic field, angle or time of flight, often considered as a single ‘measurement’. This article serves as a reference document for users of the software, and outlines the architecture and strategy of the program. An overview of some of the features, capabilities, peripheral user modules and neutron scattering tools is presented.

## Abstract 2

This article describes the *GRASP* software package, designed for the graphical inspection, reduction and analysis of multidetector data produced by the small-angle neutron scattering instruments at the Institut Laue–Langevin and other neutron sources.

## 1. Introduction

Small-angle neutron scattering (SANS) is one of the mainstay techniques of most neutron sources for materials investigation in the size range of ∼10–10 000 Å. The applicability of the SANS technique in material structure determination is broad, covering scientific domains from solution scattering in biology and soft matter to physics, materials science and magnetism. Neutrons scattered by dilute and uniformly shaped particles in solution or dispersed in a material matrix allow the determination of the particle-shape function or form factor, particle-size distributions, composition and concentration. Concentrated or ordered material structures also provide information concerning particle–particle interactions, crystallographic order, orientation and alignment through the structure and form factors extracted from the recorded scattering pattern (Grillo, 2008 ▸; Hollamby, 2013 ▸).

The Institut Laue–Langevin (ILL) hosts a suite of three SANS instruments, D11, D22 and D33, each with vast overlap in application and capabilities but with some specificities leading to a small bias in the typical science domains, with user communities preferring access to a particular instrument. The requirements for data treatment, however, remain the same. These are the ability to subtract background contributions and unwanted scattering, correct for attenuation (transmission) due to absorption and scattering, eliminate (mask) areas of the multidetector, and calibrate into real units of scattering cross section, *e.g.* barns per molecule or, more often in the case of SANS, scattering cross section per unit volume in units of cm^−1^ per steradian. In solution scattering, the pattern recorded on the 2D detector is usually isotropic around the direct beam direction with no orientation information. Data of these kind are usually reduced from a 2D scattering pattern into a 1D curve of cross section (intensity, *I*) versus scattering vector magnitude, |**Q**| = *Q*. While a single measurement may constitute all of the above, an experimental investigation usually involves the measurement of many such data, typically as a function of changing material parameters such as concentration, composition or hydrogen–deuterium ratio in contrast matching experiments, or as a function of external sample-environment parameters such as temperature, magnetic field, pressure, sample orientation, strain or sheer. An experimental investigation, therefore, more often than not involves the cross referencing and co-analysis of many individual data sets.

Isotropic scattering data and the reduction and analysis of 1D scattering curves are by no means exclusive to the broad scientific domains addressed using SANS. Anisotropic scattering and (Bragg) diffraction at small angles are often encountered in studies of hard materials, magnetic scattering (Honecker *et al.*, 2013 ▸), vortex lattices in type-II superconductors (Cubitt *et al.*, 1993 ▸; Levett *et al.*, 2002 ▸), magnetic spin structures in heli-magnetic and skyrmion materials (Mühlbauer *et al.*, 2009 ▸; White *et al.*, 2018 ▸), and induced anisotropy in, for example, sheared polymers (Porcar *et al.*, 2004 ▸) and microfluidic flow channels (Fischer *et al.*, 2023 ▸). Even classical SANS studies of solution scattering can present anisotropic scattering patterns due to highly monodisperse particle distributions at high concentrations leading to crystallinity (Hellweg *et al.*, 2000 ▸) and even quasi-crystallinity (Förster *et al.*, 2007 ▸). An in-depth analysis of these classes of data requires numerical operations and data analysis on the full 2D multidetector data, and in most cases as a function of a third experimental sample-environment parameter. *GRASP* was developed specifically for data treatment and analysis of these types of scientific problems (Dewhurst, 2001 ▸).

The majority of SANS instruments at continuous neutron sources such as nuclear reactors [with the notable exception of SINQ, Paul Scherrer Institute (PSI)] operate in a quasi-monochromatic mode with a discrete ‘monochromatic’ band of wavelengths chosen by a mechanical velocity selector. Monochromatic instruments typically count scattered neutrons for a given duration, resulting in a single 2D ‘image’ array of data representing the measured counts in the individual detector pixels over the 2D detector array. On the other hand, a different class of SANS instruments operating in a time-of-flight (TOF) mode using a pulsed ‘white’ beam are typically found at spallation neutron sources (Heenan *et al.*, 1997 ▸, 2006 ▸) as well as some at reactor sources (Kampmann *et al.*, 2006 ▸; Dewhurst *et al.*, 2016 ▸; Sokolova *et al.*, 2019 ▸). TOF instruments index the incoming neutron wavelength by the time of arrival of neutrons at the detector and, in the case of neutron instruments designed for structural material investigations, typically ignore effects of inelastic energy or wavelength change by interaction with the sample. When TOF data are histogrammed in slices of time of arrival (or wavelength), this third dimension also needs to be considered in the data treatment. The additional third dimension available in *GRASP* lends itself usefully to the treatment and analysis of time (wavelength)-histogrammed TOF SANS data.

Software tools such as *GRASP* are key in efficiently processing and cross referencing large quantities of scattering data, and offer a ‘toolbox’ of utilities to optimize extraction of the quantities of interest. This article gives an overview of the *GRASP* software for SANS data treatment and celebrates 22 years since first developments were presented to the user community.

## 2. *GRASP* history and software package

Since its first release in 2001, *GRASP* has attracted significant attention and support, both within the user community and from facility instrument responsibles of similar multidetector-based instruments at other neutron facilities, X-ray facilities and some laboratory-based X-ray small-angle scattering instruments. Some of these instruments are catered for within *GRASP*, including instruments at the ILL; SINQ, PSI, Switzerland; Helmholtz-Zentrum, Germany; Laboratoire Léon Brillouin, France; Oak Ridge National Laboratory, USA; NIST Centre for Neutron Research, USA; the Australian Nuclear Science and Technology Organisation, Australia; and Maier-Leibnitz Zentrum, Germany. Modern graphical-user-interface-based packages such as MATLAB (MathWorks, 2023 ▸) offer a high-level and system-independent computing language with easy matrix manipulation, comprehensive graphics and interface-building tools. The architecture and configuration flexibility of *GRASP* combined with the continuously developing capabilities of the professionally maintained MATLAB environment have allowed continued development of *GRASP*, with it undergoing approximately ten major architectural overhauls over its 22-year lifetime. MATLAB can be compiled into standalone licence-free runtime code for Windows, Linux and Macintosh OSX platforms, allowing *GRASP* to be distributed to users freely without obligation to buy the MATLAB package. On the other hand, many users choose to use the source m-code for *GRASP* directly in MATLAB, contributing to bug fixes, customized analysis routines (Holmes, 2014 ▸) and incorporation of user modules. The source m-code and compiled packages are freely available and distributed via the *GRASP* website hosted by the ILL at https://www.ill.fr/grasp/.

## 3. Worksheets, interfaces and layout

The internal data-storage architecture of *GRASP* is based around the concept that a single measurement should allow for 3D data: two spatial dimensions of the 2D multidetector(s) and a third dimension to be used for series data of varying sample, environment parameter or TOF. This third dimension, or ‘Depth’ in *GRASP*, is somewhat akin to image frames of a movie. Three-dimensional worksheets exist for each of the scattering data components, such as sample scattering, empty-cell scattering, transmission measurements *etc*., with typical (system-memory-dependent) limits being several thousands of data files within a worksheet ‘Depth’. Data treatment and correction for the various background contributions, calibrations *etc*., are performed directly on the 2D multidetector data on a pixel-by-pixel basis within each 2D frame (depth). The main display of *GRASP* is therefore a WYSIWYG (what you see is what you get) live display of the 2D data treatment in progress and is continuously refreshed with an up-to-date calculation of the 2D result ‘on the fly’ with every relevant operation within *GRASP*. The various data reduction, analysis and extraction tools work directly on this live 2D data treatment in extracting scattered intensities as a function of the desired parameter or dimension.

### 3.1. Main interface

**Figure 1:**
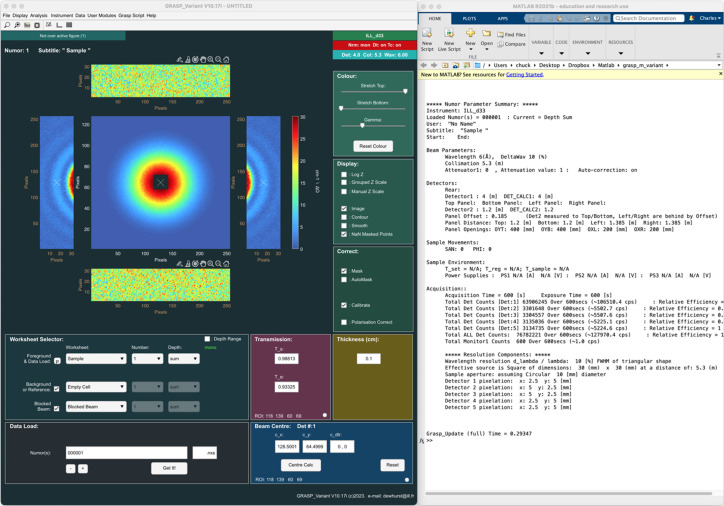
Main *GRASP* interface and command/output window.

Fig. 1 ▸ shows the main interface of *GRASP* on the left alongside the MATLAB command/output window or the text output window for the compiled version of *GRASP* on the right. The main interface hosts the 2D graphical display of data and principal GUI control elements while the output window provides a detailed text update of parameters and procedures executed during the data treatment. The 2D displayed image properties are controlled by GUI elements on the upper-right-hand side of the main interface such as logarithmic intensity display, grouped and manual intensity scale, rendering style, contour, and colour-map options. Data entry into *GRASP* and control of the displayed scattering data and background subtractions are achieved using the worksheet selectors below the main display. Data are loaded into the worksheet indicated by the ‘Foreground & Data Load’ selector with numerous options available to sum or concatenate series data as determined by the syntax of the data-load string. The data selectors allow access to a large array of workspace storage areas indexed by ‘Worksheet’, ‘Number’ and ‘Depth’. The ‘Worksheet’ selector indexes generic classes of data such as sample scattering, backgrounds, transmission and beam measurements, *etc*., while the ‘Number’ selector allows for different instrument configurations such as detector and collimation distances determining the instrument’s *Q* range. The ‘Depth’ property allows many individual measurements, for example on the same sample but with varying experimental conditions, to be collected together but stored under a single worksheet number. Whole sequences of data constituting a larger measurement can hence be analysed easily through the worksheet depth using the various reduction tools, for example extracting intensities within regions of interest (ROIs) of the multidetector (boxes, sectors and strips) or reduction to 1D *I* versus |**Q**| or scattering angle |2θ|.

Depending on the chosen foreground worksheet type, the lower two worksheet selectors ‘Background or Reference’ and ‘Blocked Beam’ perform different roles. For example, when the main selector shows sample-scattering data, the lower selectors propose worksheet types suitable for the subtraction of background-scattering contributions. When the main selector shows sample-transmission data, the lower selectors offer the reference transmission measurement and allow the sample-transmission value and beam-centre position to be calculated through the worksheet depth and then displayed in the lower right of the main display. By default, the worksheet number, depth, beam centre, transmissions and sample thickness are grouped such that scrolling from worksheet number 1 to worksheet number 2 similarly updates all the other properties corresponding to worksheet 2. Likewise, scrolling through the foreground depth of a worksheet also scrolls through the relevant depth properties of background, beam centre, transmission and sample thickness. The net result is a number of linked worksheet containers capable of storing and treating all the scattering and background data and ancillary parameters with the third dimension available for parametric analysis across a measurement series or TOF. The right-hand side of the *GRASP* main interface hosts a selection of options for controlling the 2D data treatment such as the masking of areas of the detector, enabling access to the calibration options and access to options for the treatment of polarized neutron data. Right-clicking on many of the checkboxes, push buttons or edit boxes enables rapid access to additional options otherwise found within the main menus. The command/output text window provides a live and detailed summary of the latest analysis processes, and reporting of a great many instrument-, data- and treatment-related parameters. Running live under MATLAB, the command window also serves for custom access to *GRASP* raw and treated data as well as program configuration variables, and allows the development of custom user operations. The command/output window is an incredibly valuable tool and is useful to keep visible at all times during operation of *GRASP*.

The main interface features of *GRASP* are summarized as follows:

(*a*) 2D graphical display – display of raw, corrected or calibrated detector(s) data depending on the program state and the selected normalization, correction and calibration options through the data-treatment process.

(*b*) Worksheet selectors – allow the selection of up to three components of scattering [sample scattering (‘Foreground & Data Load’); empty-cell, sample-holder or buffer-solution scattering (‘Background’); and extraneous scattering (‘Blocked Beam’)] as well as the selection and treatment of other measured data components such as transmissions, beam centres, detector efficiency *etc*. By default, worksheet number, depth, transmission and beam-centre values are grouped together, but they can be un-grouped or locked in ‘expert mode’.

(*c*) Data load. The ‘Foreground & Data Load’ selector also serves as the indicator as to where loaded data should be placed within the data-storage arrays. Measurement-run numbers must contain an incremental numeric part in the file name, as well as any pre- or post-numeric static parts in the file name and data extension. Sequences of data may be loaded in various schemes, as defined by the input syntax. The various loading options conserve or accumulate normalization parameters such as acquisition time, beam-monitor statistics *etc*., as appropriate.

(*d*) Transmission – display, calculation tools, or manual entry of sample and empty-cell transmissions.

(*e*) Thickness – display or manual entry of the sample thickness.

(*f*) Beam centre – display, calculation tools or manual entry of the current beam-centre coordinates.

(*g*) Display control – colour scheme, render, contour options, smoothing, intensity scaling, axis and intensity range.

(*h*) Masking, calibration, polarization and analysis corrections – shortcuts to opening and activating principal data-correction options (otherwise available in the main menu).

### 3.2. Spreadsheet interface

**Figure 2:**
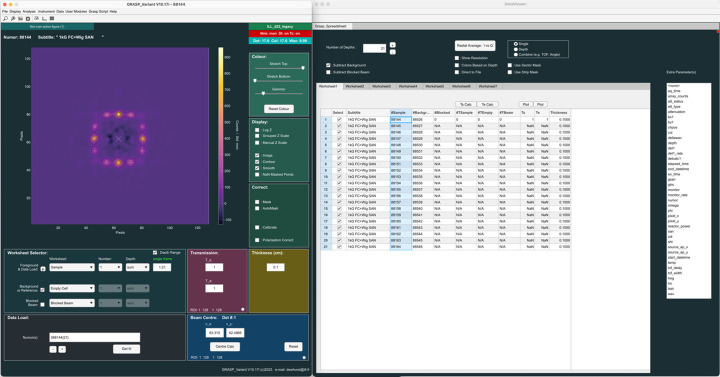
Alternative *GRASP Spreadsheet* interface.

An additional spreadsheet interface can be opened to facilitate navigation and selection of data through the various worksheets, and to perform the most common data reduction tasks, as shown in Fig. 2 ▸. The spreadsheet presents columns indicating the sample-scattering run number and title, as well as those for the background, blocked beam and transmission measurements. Derived quantities such as the sample and empty-cell transmission values are also shown, while a number of additional parameters such as the varying parametric variables through the worksheet depth can be selected to be displayed alongside the data columns. The columns show data through the worksheet depth, while the worksheet number itself is selected by the tabs at the top of the spreadsheet. The main *GRASP* interface and the spreadsheet interface are completely inter-operable, with the program status updated regardless of the interface used.

### 3.3. Menu and toolbar features

The menu and tool items in the main *GRASP* interface allow access to control program behaviour, input–output, instrument and data configurations, and the wide variety of data extraction and reduction and analysis modules. The main-menu features of *GRASP* are summarized as follows, while some of these items can also be found in the toolbar, main and spreadsheet interfaces:

(i) File – general project tools including loading/saving *GRASP* projects, definition of data sources, import and export of data, masks, detector efficiencies, print and export of images, and program preferences.

(ii) Display – control and options for the main 2D display, *GRASP* interface and text output window including image-display render, colour maps and palette manipulation, contour options, display options, and axis options.

(iii) Analysis – access to the various data-extraction toolboxes and interfaces including the *GRASP* spreadsheet interface; radial and azimuthal averaging tools; magnetic scattering analysis; box, sector and strip ROIs; 2D curve fitting; mask editor; calibration options; detector-efficiency calculator; and polarization and analysis tools. The data reduction algorithm may also be specified in the analysis menu, defining how operations are performed between the data selectors.

(iv) Instrument – choice of instrument and instrument configuration file, instrument viewer, and the SANS instrument simulation tool.

(v) Data – raw-data and metadata inspection tools, parameter survey, parameter patch, data-correction and normalization options, detector deadtime, transmission thickness, and attenuator corrections. The resolution control centre allows users to specifically tailor and inspect contributions to the overall calculated instrument resolution δ*Q*(*Q*).

(vi) User modules. The already comprehensive suite of data-inspection and analysis tools can be enhanced or customized by the user contribution of user modules, fitting functions or *GRASP* script routines. Submitted and validated user modules are available under the user-modules menu, with current science-specific examples being tools for the analysis of diffraction from the vortex lattice in type-II superconductors; a rheological scattering anisotropy calculator; plotting, extraction and analysis of parameters from non-SANS *in situ* techniques; and SANS instrumentation calculators such as the TOF calculator and the D33 chopper time–distance calculator.

(vii) *GRASP Script* – allows access to the *GRASP Script Editor*, running of *GRASP* scripts and *GRASP Script* help. *GRASP Script* allows MATLAB and *GRASP* functions to be executed as a script of commands in order to automate data processing in *GRASP*. Scripting and execution of MATLAB commands are even possible in the compiled version exploiting the eval functionality of the software. Automation is easily achieved using standard MATLAB commands, via a dedicated set of macro-commands using the gs command, and through the globally accessible variables parameterizing the interface features and data-treatment options.

(viii) Help – access to documentation including the *GRASP* manual, notes on polarization and analysis corrections, and *GRASP* developer notes.

## 4. Data treatment and scattering geometry

### 4.1. Data correction and absolute intensity

Primary data treatment in *GRASP* proceeds by preserving the 2D nature of each detector image or frame including series or TOF data through the worksheet depth. Data treatment and correction is made on a pixel basis and is continually updated on the fly with every relevant operation in *GRASP*, with the result shown in the main 2D display. The internal data storage always holds a copy of the raw experimental data, *i.e.* neutron counts, along with the relevant metadata such as sample information, thickness, instrument geometry, counting time, beam reference monitor, attenuation status *etc*., in order to enable full geometric correction and intensity calibration of the data. Statistical errors associated with the raw counts are generated according to Poisson counting statistics for random events as the square root of the raw number of counts in a pixel, and are used for subsequent error propagation throughout the data treatment and regrouping analysis procedures. Custom operators for common mathematical functions such as addition, subtraction, division, multiplication, trigonometric functions *etc*., exist as methods within a custom error-data class definition in *GRASP* to aid mathematical operation and the propagation of errors through the data treatment and reduction process. The standard data-treatment process can be summarized as follows, with worked examples available on the *GRASP* website (https://www.ill.fr/grasp/).

(1) Load data into the *GRASP* workspaces. Scattering-component data, *e.g.* from the sample, *I*_s_, empty cell (or *e.g.* buffer solution), *I*_bck_, and blocked beam, *I*_Cd_, measurements, are loaded into the pre-allocated scattering worksheets. Transmission-component data for the sample, *I*0_s_, empty cell (or *e.g.* buffer solution), *I*0_bck_, and empty beam, *I*0, measurements are loaded into the transmission worksheets. These data should contain all the necessary metadata such as sample information, thickness, instrument geometry, counting time, beam reference monitor, attenuation status *etc*., to enable a full calibration of the data.

(2) Deadtime correction. All data are corrected for detector-deadtime effects and treated on a per-detector basis, *i.e.* over the entire detector for multiwire detectors or on a tube-by-tube basis for a multitube detector type. Data are corrected on the basis of the non-paralyzable model for a Poisson statistical process as *n* = *m*/(1 − *τm*), where *n* is the real count rate, *m* is the measured count rate and τ is the deadtime (Pritchard *et al.*, 2021 ▸). The instrumental deadtime should be provided by the facility instrument responsible, upon establishing configuration of the instrument in *GRASP* (Section 6.4).

(3) Normalization and data scaling. All data are normalized to the measurement time or exposure to the neutron beam by normalization to either acquisition time or the beam monitor, to enable comparison of the various data that may have been acquired for different times or to consider variations in the incoming intensity from the neutron source. Transmission or beam measurements made with an attenuator placed in the beam are upscaled by the attenuation factor.

(4) Masking. Unwanted or bad regions of the 2D detector data are masked and removed from taking part in the analysis using the mask-editor tools.

(5) Transmissions. The transmission of the sample, *T*_s_, is calculated relative to that of the empty cell (or *e.g.* buffer solution) as *T*_s_ = *I*0_s_/*I*0_bck_. The transmission of the empty cell, *T*_e_, is calculated relative to that of the empty beam as *T*_e_ = *I*0_bck_/*I*0. An ROI around the beam on the detector can be selected to eliminate unwanted scattering from the transmission measurement. For monochromatic measurements, a single transmission value is applied to the scattering data. For TOF data, each wavelength-histogrammed slice in the worksheet depth is accompanied by a wavelength-dependent transmission value also calculated with associated uncertainties through the transmission worksheet depth.

(6) Direct-beam intensity. The empty-beam measurement, *I*0, also serves as a reference of the incoming (attenuated) beam intensity of the measurement and as the reference beam centre of mass, Cm_*xy*_ (the coordinate centre of scattering), for the scattering data. An ROI around the beam on the detector can be selected to eliminate unwanted scattering from the beam-centre measurement.

(7) Correction for backgrounds and absorption. Enabling the subtraction of empty-cell and blocked-beam scattering contributions removes these background contributions from the sample-scattering data, while taking into account the transmission of the sample and the empty cell. By default, the correction of background scattering proceeds as

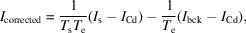

and assumes that the transmission values estimated for the sample and the empty cell are due to absorption effects, and not due to forward beam attenuation caused by strong scattering. Other data-correction schemes, such as the treatment of polarization and analysis data (Section 6.1), or non-standard experiment-specific schemes, such as the over-illumination of microfluidic sample cells, may be requested or added by users and made available in the analysis menu.

(8) Calibration to units of differential cross section, dσ/dΩ. Enabling the calibration options allows the corrected scattering intensity to be calibrated directly into units of differential cross section, dσ/dΩ. Traditionally, for SANS, this is usually expressed as a differential cross section per unit volume in units of cm^−1^ per steradian. The calibration options in *GRASP* involve (*a*) normalization to the direct-beam flux (*I*0/cross-sectional area), (*b*) correction for fluctuations in detector efficiency, (*c*) correction for relative detector efficiency between multiple detector banks, (*d*) parallax correction (detector-type specific), (*e*) division by pixel solid angle subtended on the sample (dΩ), (*f*) path-length-dependent transmission correction and (*g*) division by sample volume (cross-sectional area multipled by sample thickness, *t*). In the simplest case, and ignoring the additional detector efficiency and parallax flat-field corrections, the final scattering cross section can be expressed as

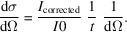




### 4.2. Scattering geometry

At the very heart of *GRASP* is the calculation of the scattering geometry for every detector pixel, referred to within *GRASP* as the *qmatrix*. The *qmatrix* is a 3D matrix with the first two dimensions matching those of the detector data and the third dimension indexing for every detector-pixel quantity, such as pixel coordinates (*x*, *y*), scattering vector components (|**Q**|, *Q_x_*, *Q_y_*, *Q_z_*), scattering angle (|2θ|, 2θ_*x*_, 2θ_*y*_), azimuthal angle (ψ), solid angle (δΩ), radial distance (|*r*|, *r_x_*, *r_y_*) and the resolution components described below. The *qmatrix* is calculated from the detector pixelation for either flat-panel or 1D-curved (banana) shaped detector geometries, along with the associated instrument motor positions such as sample and detector positioning and any detector translation or rotation. Calculation of the various geometric quantities is in principle rather straightforward but, in reality, can involve some careful and detailed geometric calculations depending on the instrument geometry, motorized axes, centres of rotation and detector curvature. As for the corrected intensity, the *qmatrix* geometric components are recalculated on the fly with every update of the scattering data, or derived parameters such as beam centre, and form an integral part of the more general grasp_update refresh routine. All 2D scattering data therefore carry with them this *qmatrix* of geometric parameters defined by the scattering geometry and instrument parameters, and calculated as if at the centre of each pixel.

### 4.3. Instrument resolution

Each pixel of the 2D multidetector data records the scattered intensity at its nominal scattering vector magnitude, |**Q**|, and scattering vector components, *Q_x,y,z_*, as smeared by the various instrument-resolution contributions. In other words, the intensity measured by each pixel is the (weighted) sum of scattering within a finite window of scattering vector magnitudes, often characterized by a standard deviation |σ_*Q*_| or components σ_*Q*___*x,y,z*_, and an assumed Gaussian weighting function. Metadata carried with each file should contain all the information required to be able to estimate the distribution and weight of the various instrumental (and data processing) components that contribute to the overall resolution function describing the scattering vector magnitude. For SANS data from a monochromatic instrument, a given pixel of data may carry resolution components of:

(i) Wavelength spread, σ_*Q*___λ_, with a triangular weighting distribution (for a velocity selector) and in a radial direction from the beam centre.

(ii) Geometric beam divergence. The geometric beam divergence is the spread in the incoming-beam direction and can be characterized by two separate angular contributions to the scattering vector magnitude, σ_*Q*___source_ and σ_*Q*___sample_, describing the contributions of the source and sample apertures separated by the collimation length. These contributions can be either calculated from the instrument geometry or extracted directly from the direct-beam measurement.

(iii) Detector pixelation. Detector pixelation gives an additional uncertainty in the scattering angle and therefore scattering vector magnitude, σ_*Q*___pixel_, of a detected neutron due to the angular size of a pixel for a given detector distance. Pixelation smearing can be calculated directly from the detector-pixel size (usually) in Cartesian coordinates and can be resolved into the radial direction.

(iv) Sample thickness. Sample thickness can give an appreciable uncertainty in the effective detector distance for a given scattering event and contributes to an uncertainty in scattering vector magnitude, σ_*Q*___thickness_.

(v) Software regrouping of data. Software regrouping of data into ‘bins’ with a finite span of scattering vector magnitude also contributes to the smearing, σ_*Q*_bin_, of reduced scattering data.

*GRASP* carries the various resolution components and approximate shape functions describing those components in both Cartesian and radial coordinates for each detector pixel of the 2D image. In the simplest or ‘classic’ estimate of the instrument resolution, the various resolution contributions are assumed to be Gaussian with standard deviations and are added in quadrature to give an estimate for the final resolution σ_*Q*_ (Pedersen *et al.*, 1990 ▸). A more complete method of calculating the instrument resolution is to use the component-smearing-function shapes and widths directly to numerically evaluate the final ‘real-shape’ resolution (Nelson & Dewhurst, 2014 ▸). This is the default method within *GRASP*. Under regrouping of data, the real-shape resolution kernels are also averaged to give the final resolution or smearing function describing the data in each 1D *Q* bin. At present, this real-shape kernel exists only internally within *GRASP* and is used in subsequent data fitting within the program. This is primarily because of the lack of software within the small-angle scattering community that can deal directly with the more detailed real-shape kernels. Upon export of the 1D reduced *I* versus |**Q**| data, the real-shape resolution kernel is reduced into standard deviation with an assumed Gaussian profile for simplicity and compatibility with other existing codes. TOF data are treated in *GRASP* as a series of monochromatic frames, with the instrument resolution calculated for each frame as described above. The resolution of the final re-binned TOF data is then a weighted sum of the individual resolution kernels at a given point in |**Q**|. The resolution control centre gives detailed control over the resolution calculation in *GRASP*, while the real-shape resolution kernel information for regrouped points in |**Q**| is used in scattering-model smearing for curve fitting within *GRASP* or exported as a final sigma in exported 1D data.

### 4.4. Data-reduction and -extraction tools

All data reduction in *GRASP* begins with the creation of a 2D mask of valid data points, as defined by the various selection tools such as masks, strips, boxes or sectors. Once defined, the valid pixels are extracted along with their associated geometric quantity from the *qmatrix* forming a list of pixel intensity, error and resolution components versus geometric parameter, *e.g.* scattering vector magnitude |**Q**|, scattering angle |2θ| or azimuthal angle around the detector ψ. The list of valid pixel intensities and geometric parameter is then re-grouped, averaged or re-binned, as defined by the various module re-grouping parameters. The above procedure can be carried out automatically on a single worksheet depth, carried out individually for many worksheets through the depth or re-combined through the entire worksheet depth, as appropriate for TOF data.

Data-extraction tools, such as ‘Projections’, ‘Strips’ and ‘Sectors’, allow the extraction of ROIs from the 2D detector (through the worksheet depth) as a function of scattering geometry, *e.g.* |**Q**|. Tools such as ‘Boxes’ and ‘Sector Boxes’ define ROIs, allowing parametric extraction of the integrated or average intensity over an ROI on the 2D detector as a function of data parameters such as sample angle, temperature, magnetic field *etc*.

## 5. Data-treatment examples

In this section, three worked examples are presented highlighting the principal functionalities of *GRASP*, with the step-by-step worked examples available for download on the *GRASP* website.

### 5.1. Monochromatic and isotropic data reduction, instrument resolution, and curve fitting

**Figure 3:**
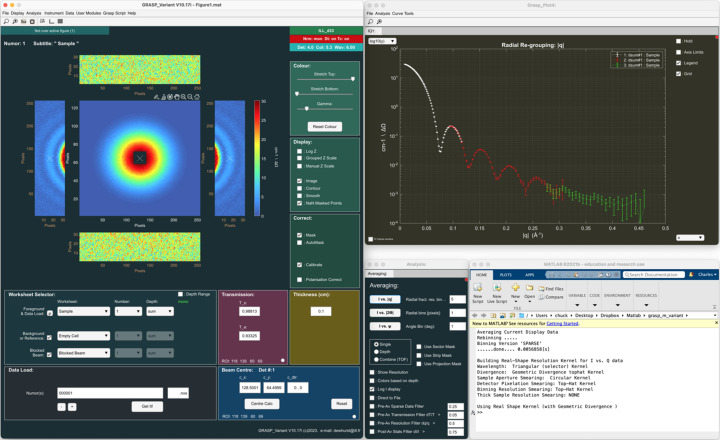
(Simulated) scattering from monodisperse spherical particles in D_2_O on the D33 instrument at the ILL. The data were simulated using the ‘SANS instrument model’ available in *GRASP* and include the effects of background scattering and instrument resolution. The reduced *I* versus |**Q**| plot shows data from the rear (white points), left–right (red points) and top–bottom (green points) detector panels.

Fig. 3 ▸ shows an example of data treatment for isotropic solution scattering of spherical particles in a solution of D_2_O. In this example, the data have been simulated for the D33 instrument at the ILL using the ‘SANS instrument model’ (Section 6.3) available in *GRASP*, and the effects of background scattering from a D_2_O buffer within a quartz cell and the blocked beam are included. The reduction of the transmitted beam intensity due to scattering is accounted for and the data contain statistical variation according to Poisson statistics, and they are scaled realistically to match the real intensity of the D33 instrument at a nominal reactor power of 55 MW. In this example, the sample was simulated as monodisperse spherical particles in solution of radius 60 Å, contrast of 6 × 10^−6^ Å^−2^ and with a fractional concentration of 0.01. The simulated instrument was set up with an incoming wavelength of 6 Å and a beam collimation of 5.3 m, and with the two detector trolleys at 4 m (rear central detector) and 1.2 m (front panel detectors). Importantly, the ‘SANS instrument model’ also reproduces the effects of instrument resolution.

Fig. 3 ▸ shows how the main *GRASP* interface is set up with all background components subtracted, transmissions and beam centre calculated, and calibration options enabled, as described in Section 4.1. The ‘Averaging’ analysis module has been opened, allowing the user to produce the final 1D isotropic scattering curve in absolute units of cross section, as indicated by the output ‘GRASP_Plot’ window. The white points show data from the rear detector, while the red and green points show data from the side and top/bottom front panel detectors, respectively.

**Figure 4:**
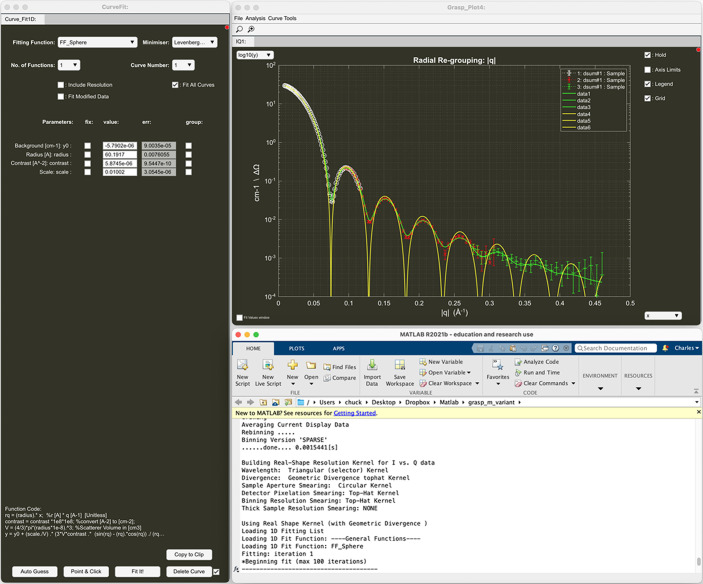
Fitting of the 1D *I* versus |**Q**| reduced data in Fig. 3 ▸ using the ‘Curve Fit’ module. The data are fitted to the same spherical-particle model and are shown both including (green line) and without (yellow line) convolution to the instrument resolution in order to achieve a satisfactory fit to the data.

Fig. 4 ▸ shows the fitting of the reduced 1D scattered intensity to the same spherical-particle model used in the instrument simulation. The 1D curve fit module can be opened from the ‘GRASP_Plot’ menus, allowing least-squares fitting of data to various user-defined model functions. The ‘GRASP_Plot’ window in Fig. 4 ▸ shows the result of the fitted spherical-particle model function, both including (green line) and without (yellow line) the convolution with the instrument-resolution function enabled in the ‘Curve Fit’ module window, along with the final fitted model parameter values. Users of *GRASP* are able to add their own analytical or numeric fitting functions of MATLAB code to *GRASP* in the functions1d.fn file following the examples already included, and they can even be edited and executed in the compiled version of *GRASP*.

### 5.2. TOF data reduction

**Figure 5:**
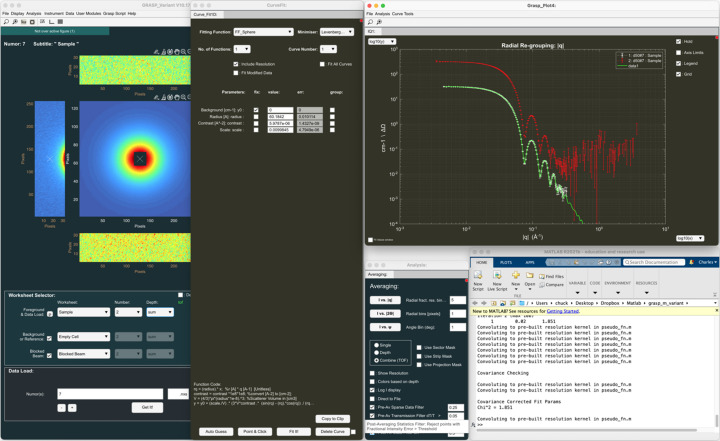
Treatment of TOF data in *GRASP* for the same simulated sphere form-factor sample described in Section 5.1. The final *I* versus |**Q**| is extracted from many time frames stored in the worksheet depth through the wavelength band. The red points show all data (×10) through the TOF spectrum, while the white points and model fitting (green line) show TOF data to eliminate data of poor statistical quality or resolution.

Fig. 5 ▸ shows the treatment of TOF data in *GRASP* for the same simulated sphere form-factor sample as described in Section 5.1. *GRASP* loads time (wavelength)-histogrammed TOF data as individual time- or wavelength-sliced data into the worksheet depth and thus appears as a sequence of quasi-monochromatic images through the worksheet depth. Users can scroll through the individual wavelength frames in two dimensions in order to visually inspect the data as a function of wavelength and perform elimination or individual masking of frames to remove unwanted features such as spurious Bragg scattering from the instrument windows at short wavelengths. The final *I* versus |**Q**| shown in the ‘GRASP_Plot’ window can be extracted in a similar manner to the monochromatic case using the ‘Averaging’ analysis tool. Here, options exist to define which range of wavelength data to include in the averaging procedure, as well as automated filtering options to eliminate sparse data, statistically poor data or data of poor resolution from the final *I* versus |**Q**|. The ‘GRASP_Plot’ window in Fig. 5 ▸ shows the *I* versus |**Q**| curve extracted over all detectors using the full wavelength range with no filtering (red curve) and that extracted by limiting the wavelength range to where statistically relevant using the filtering options (white curve). A particular scattering vector magnitude, |**Q**|, appears at a different position on the detector (scattering angle) as a function of wavelength and therefore with different instrument resolutions. This is where the importance of the numerical kernel method should be highlighted, as not only do the different wavelength contributions to a particular |**Q**| have different instrument resolutions, but these resolution components can also have very different kernel shapes. For example, on the D33 instrument in TOF mode (Dewhurst *et al.*, 2016 ▸), the optically blind double-chopper system results in a ‘top hat’ or square wavelength resolution profile, in contrast to the triangular (close to Gaussian) profile produced by a velocity selector that dominates the instrument resolution at large scattering angles. The extracted *I* versus |**Q**| and fit of the scattering model to the data including the instrument resolution demonstrate the powerful estimation of the overall instrument resolution for TOF data using the weighted average numerical kernel method.

### 5.3. Anisotropic data treatment, 2D fitting and parametric analysis

**Figure 6:**
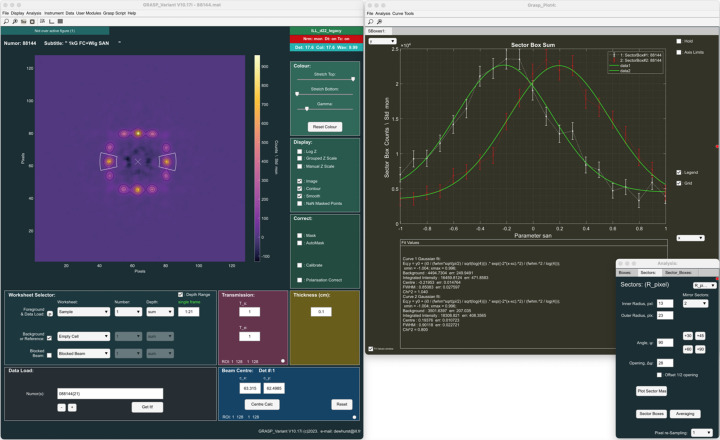
Bragg peaks from the vortex lattice in the type-II superconductor YNi_2_B_2_C (Levett *et al.*, 2002 ▸). Using the ‘Sectors’ and ‘Sector Boxes’ analysis tools, rocking curves can be extracted as the sample is rotated through the Bragg condition. The rocking curves can be fitted using the 1D curve fit tools to extract fundamental physical properties of the superconductivity.

Fig. 6 ▸ shows Bragg peaks from the magnetic vortex lattice in the type-II superconductor YNi_2_B_2_C (Levett *et al.*, 2002 ▸), as measured on the D22 instrument at the ILL. The 2D image presented in the main *GRASP* interface is in fact the sum of 21 individual measurements as the sample and magnet are rotated in the horizontal plane through the Bragg condition. Twenty-one background measurements can be subtracted frame by frame from the sample-scattering data. This sequence of both sample and background parametric measurements as a function of sample angle is collected together in the *GRASP* worksheet depth. Using the ‘Sectors’ and ‘Sector Boxes’ analysis tools, an ROI over the principal Bragg peaks in the horizontal plane can be defined and scattered intensity of the Bragg peak extracted as a function of the sample-angle parameter, as shown in the ‘GRASP_Plot’ window. The rocking curves can be fitted, for example with a Gaussian function using the ‘Curve Fit’ tool, to extract the integrated intensity and rocking-curve width, which are in turn directly related to the fundamental physical properties of the superconductor, the magnetic form factor and the longitudinal correlation length of the vortex lattice.

**Figure 7:**
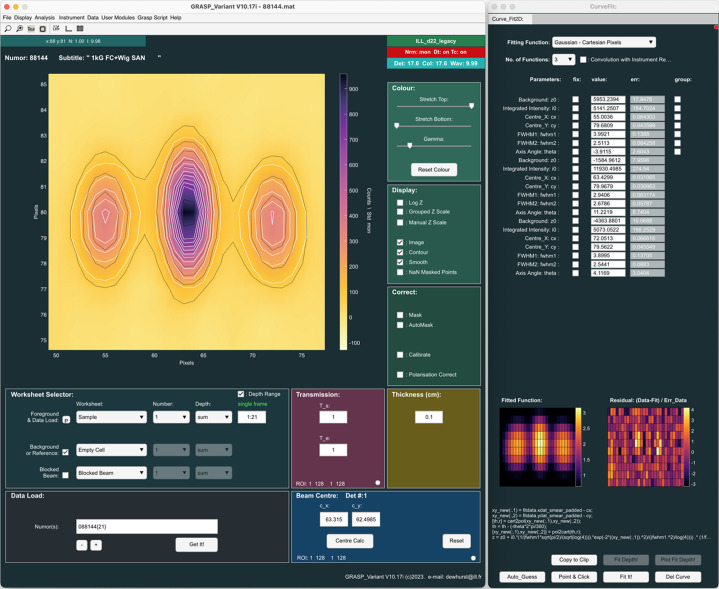
Simultaneous fitting of Bragg peaks in the 2D data from the vortex lattice in YNi_2_B_2_C, summed over the rocking curve (Levett *et al.*, 2002 ▸).

The 2D curve fitting module allows multiple simultaneous functions to be fitted directly to the corrected multidetector data, as shown in Fig. 7 ▸. Here, three simultaneous 2D Gaussian functions are fitted to the top three Bragg peaks of the summed rocking-curve image. The 2D curve fitting tools allow for the grouping of common parameters for multiple function fits or the fixing of parameters. The example in Fig. 7 ▸ shows the fitting of three simultaneous 2D Gaussian functions in pixel coordinates on the 2D detector data, while fixing the orientation of the Gaussian spots at 0° and constraining the widths of the spots in the horizontal and vertical directions to be the same. The ‘2D Curve Fit’ module shows the resulting fit parameters, a graphical representation of the 2D fitted functions and the residual (data minus the fit divided by the statistical error in the data). Two-dimensional fitting functions can be included by users in the functions2d.fn file, and they can even be edited and executed in the compiled version of *GRASP*.

## 6. Other tools and capabilities

A brief summary of some of the other *GRASP* capabilities is presented in this section.

### 6.1. Polarization and analysis

Dedicated worksheets exist within *GRASP* to cater for the requirements for half-polarization (SANSPOL) with ‘+’ or ‘−’ incoming spin states, and for full polarization and analysis of the scattered-beam polarization (*i.e.* ++, −−, +− and −+, denoting the incoming and scattered spin state) (Honecker *et al.*, 2013 ▸). Usually, the incoming-beam polarization is defined by a solid-state mirror polarizer and adiabatic spin flipper. Polarizer and spin-flipper efficiencies are measured experimental parameters and should be adjusted during the data-treatment process in the ‘Polarisation Analysis’ modules in *GRASP*.

Analysis of the scattered neutron spin is most usefully achieved on SANS instruments using polarized ^3^He gas as a spin analyser. Polarized ^3^He gas is typically produced on-site using optical pumping techniques and can reach ^3^He gas polarizations of ∼80% (Andersen *et al.*, 2005 ▸). Polarization of the ^3^He gas is maintained on the instrument using a ‘magic box’ highly uniform magnetic field cavity, and polarization of the neutron beam can reach values much greater than that of the ^3^He gas depending on the gas pressure and cell thickness. Nevertheless, the polarization of the ^3^He analyser gas decays exponentially as a function of time (with a half-life of 10s to 100s of hours) with corresponding varying analysing efficiency. These parameters need to be regularly monitored during a polarization-analysis experiment and incorporated into the data reduction using the ‘Polarisation & Analysis’ modules in *GRASP*, which is not covered in detail in this article. The data-analysis procedures for SANSPOL and polarization analysis were implemented in *GRASP* by D. Honecker and are described in detail by Honecker *et al.* (2013 ▸).

### 6.2. GRASP_Plot

‘GRASP_Plot’ is the 1D graph output window, as highlighted in Figs. 3 ▸–7 ▸ ▸ ▸ ▸. As well as providing a standardized interface for reduced data, ‘GRASP_ Plot’ also provides a number of useful tools for manipulation of 1D data produced by *GRASP*. These are:

(1) Display options – the usual graph-inspection tools such as zooming, panning, control of axis limits, axis scaling, legends and grids.

(2) Save ‘GRASP_Plot’. ‘GRASP_Plots’ can be saved as regular MATLAB figures and reopened directly from the main *GRASP* interface or the existing ‘GRASP_ Plot’. Open ‘GRASP_Plots’ are automatically saved with the saving of a *GRASP* project from the main interface.

(3) Image export – graphical export of figures in the usual image formats such as JPG, BMP and PNG, or in vector formats such as Adobe PDF or AI (Illustrator). Bitmap images can also be copied to the clipboard to paste directly into documents.

(4) Data export and import. One-dimensional curves can be exported as ASCII data columns, including the instrument resolution or resolution components where appropriate. ASCII column data can be re-imported into ‘GRASP_Plot’ at a later stage if required.

(5) Page-layout print – a graphical summary of the current plot with a text summary of instrument parameters and data reduction history. Any current fit parameters are also reported.

(6) 1D curve fitting – curve fitting and fit-parameter analysis, as shown in the examples in Section 5.

(7) Calculate moments – elementary peak analysis without fitting based on the numerical calculation of quantities such as the sum, mean, area, centre of mass, standard deviation, skewness and kurtosis.

(8) Show/hide resolution – shows the *Q* resolution as horizontal error bars on an *I* versus |**Q**| plot as plus and minus the full width at half-maximum of the resolution function.

(9) Contrast-match module – allows the contrast-match point to be determined from a series of *I* versus |**Q**| curves at different H_2_O/D_2_O ratios.

(10) Merge curves. Separate *I* versus |**Q**| curves originating from different instrument configurations can be merged into a single curve including options for cropping, scaling and auto-scaling between curves.

(11) Mathematical operations – on curves and between curves.

(12) Curve editor – opens curve data in a curve-editor spreadsheet.

### 6.3. SANS instrument model

*GRASP* contains a rather useful data-simulation tool, the ‘SANS instrument model’, giving users of the software the ability to simulate scattering from a few typical sample and background models, for example a sphere form factor with user-definable parameters such as particle radius, polydispersity, contrast and concentration. The software also aims to reproduce most optical features of the instrument, instrument resolution, neutron flux and counting statistics in order to provide realistic looking 2D simulated data, as if it had come from the real instrument and as shown in Figs. 3 ▸–5 ▸ ▸. Simulated data can then be corrected and reduced to 1D in *GRASP* just as for real scattering data. The instrument optical model is analytic while scattering at the sample is simulated using the Monte Carlo method. Incoming-beam parameters such as the divergence profile due to source and sample apertures as well as the wavelength spread of the neutrons are modelled as weighted probability shape kernels in a method analogous to that of the treatment of resolution effects described in Section 4.3. The detection of scattering within a given detector element is calculated according to randomized distributions over divergence and wavelength spread describing the incoming-beam characteristics. The model is randomized over several iterations and scaled to absolute intensities according to the representative intensity characteristics of the instrument. Poisson noise is added to the simulated and scaled detector-pixel counts to mimic the expected statistical variation in counts. The simulated monochromatic and TOF data shown in Figs. 3 ▸–5 ▸ ▸ were generated using the ‘SANS instrument model’ for the D33 instrument with a dilute and perfect sphere form-factor model of radius 60 Å, zero polydispersity, contrast 6 × 10^−6^ Å^−2^ and concentration 1%. Background-scattering components of a 1 mm thick sample of D_2_O buffer solution were modelled on the basis of real measurements. While the methodology describing the instrument resolution as the convolution of various real-shape distributions in divergence, wavelength *etc*. is the same both for the treatment of data in GRASP and for the ‘SANS instrument model’, the precise implementation and code are different. These are effectively the reverse processes of each other. Data treatment calculates the overall resolution function for a given scattering vector magnitude based upon geometric or measured distributions of wavelength, divergence and position. However, the ‘SANS instrument model’ performs a Monte Carlo simulation of scattering at the sample, generating 2D detector data resembling what the instrument itself would measure, and based on the given sample-scattering model and the relevant distributions of wavelength, divergence and position. The simulated data shown in Figs. 3 ▸–5 ▸ ▸ therefore serve as a useful demonstration of the resolution kernel method implemented in *GRASP*.

### 6.4. Instrument configuration files and data loaders

Instrument descriptions in *GRASP* are based on text-based configuration files that are read from the instrument_ini directory and included into the *GRASP* menu of available instruments upon launching the program. New instruments can be included into *GRASP* in a relatively straightforward manner even in the compiled version of the software and do not need to be pre-compiled into the code. A minimum instrument configuration file contains information such as the instrument and facility name, a description of the data-file naming protocol, and a pointer to the MATLAB-coded data loader. The data loader can be executed even within the compiled version of the code. The detector(s) configuration and geometry are defined by the detector type (‘tube’, ‘multiwire’ or ‘banana’) along with parameters defining the number of pixels, pixel size and detector deadtime, and several default geometric parameters describing detector position, translations, rotations and offsets relative to the sample position. Default detector mask files, flat-field detector-efficiency files and relative detector efficiencies can also be specified. The maximum detector sizes that are useable within *GRASP* are essentially limited by computer memory and speed but are perfectly reasonable for detectors commonly used in neutron instrumentation. The largest detectors currently described and used in *GRASP* are those of the new D16 cold neutron diffractometer with a banana detector of 220K pixels and the D22 *in situ* small-angle X-ray scattering instrument with 1M pixels.

There are no specific requirements in terms of data formats, with ASCII, HDF, NeXuS HDF variant (Könnecke *et al.*, 2015 ▸), binary or SPE formats all commonly used in the scattering community. The only requirement is that native MATLAB routines are able to open and read the files. Often data from neutron sources contain histogrammed detector images of single measurements or time slices for TOF or kinetic data that are most easily catered for in *GRASP*. *GRASP* now offers some limited support for reading and histogramming event-mode data as new instruments, methods and data formats appear. Data-file names must have an incremental numeric part to best use the capabilities of *GRASP* for the treatment of large series of data but can contain non-incremental or non-numeric parts before and after followed by the filename extension, *e.g.* aaa012345bbb.nxs. Data-loader code must accept an input argument containing the path and filename of the data to be loaded. The output from the data loader must be a single structure-type variable with fields including the detector counts, statistical errors, and key instrument parameters such as the sample-to-detector distance and the wavelength required for the calculation of scattering vector magnitudes within *GRASP*.

Any user of *GRASP* who is interested in the addition of new instrument configurations is invited to look at existing configuration files and data loaders included in *GRASP*. For the inclusion of new instruments, instruments of more complex setups or customized data-treatment protocols, the user is invited to contact the author.

## 7. Summary

In this article, we have described the software package *GRASP* designed for the treatment, reduction and analysis of multidetector data from SANS instruments at the ILL and other neutron sources. *GRASP* has been available to the ILL and wider SANS user communities for more than 22 years. It serves as a vital tool in the process from measurement to publication, and is crucial for rapid inspection of data on the beamline during the course of experiments. The primary philosophy of *GRASP* is that the correction of data for background scattering, detector efficiencies, parallax data correction and geometric corrections is performed in two dimensions with the user aware of the 2D result at all times. Data reduction and extraction into 1D scattering curves, such as *I* versus |**Q**|, proceed directly from these 2D fully corrected and calibrated data. A second fundamental architectural feature of *GRASP* is related to the fact that an experimental investigation often may involve many individual measurements requiring a parametric analysis through, for example, changing sample concentration, hydrogen–deuterium ratio or sample-environment parameters, with data analysis often involving the cross referencing and co-analysis of many individual data sets. The ‘depth’ to a given *GRASP* worksheet number provides this additional dimension to accommodate and regroup individual measurements as part of a single experimental investigation. This additional dimension of data storage and analysis also lends itself usefully to the analysis of TOF SANS data.

*GRASP* has been developed in the high-level MATLAB programming environment, which offers a professionally maintained programming infrastructure tailored specifically to facilitate easy and rapid development of code in science, engineering and industrial contexts. Although a commercial software, licence fees remain ‘reasonable’, at least for academic use, and are in line with those of other commonly used commercial software such as for word processing and presentation. Many *GRASP* users choose to use the code directly within MATLAB and contribute user modules, bug fixes and additional functionality directly. On the other hand, MATLAB code can be ‘compiled’ (or more correctly packaged in a runtime form), allowing licence-free distribution of the runtime version of *GRASP*.

The development of *GRASP* has often been criticized in its potential for single-point failure in relying on, for the most part, a single code author (*i.e.* the author of this article) and lacking a formal project organizational structure. Up until now this has never posed a significant problem, with contingency in place in terms of freely accessible, well written, organized and commented code and developer documentation. It is this author’s opinion that, for a project on the scale of *GRASP*, the absence of a heavy software project structure allows for creativity in the development of new features and rapid response to bug reports or feature requests. The maintenance of the code has been successfully handed over to co-workers or summer students for certain periods during *GRASP*’s history, thus demonstrating that code maintenance and development by others is indeed possible. The many instrument scientists, users and summer students who have contributed or maintained the code over the years demonstrate the ease and ability for persons not familiar with the code to rapidly get up to speed. The single-author approach is not uncommon in the provision of successful scientific software (Rodríguez-Carvajal, 1993 ▸; Weber *et al.*, 2016 ▸; Qureshi, 2019 ▸; Katcho *et al.*, 2021 ▸) but goes against the growing trend of large collaborative software projects, and the benefits and inconveniences that such organizations bring (Arnold *et al.*, 2014 ▸; Wuttke *et al.*, 2022 ▸). As of March 2022, and with a reorganization of the ILL’s scientific computing group, *GRASP* is now an officially maintained ILL software program.
